# Vaccination coverage, vaccine hesitancy and factors associated with incomplete vaccination: a household survey conducted with children born between 2017 and 2018 in the inland municipalities of Northeastern Brazil

**DOI:** 10.1590/S2237-96222024v33e20231224.especial2.en

**Published:** 2025-01-10

**Authors:** Anderson Fuentes Ferreira, Alberto Novaes Ramos, Adjoane Mauricio Silva Maciel, Jaqueline Caracas Barbosa, Ramon da Costa Saavedra, Maria Bernadete de Cerqueira Antunes, Luisa Helena de Oliveira Lima, Rejane Christine de Sousa Queiroz, Taynara Lais Silva, Martha Suely Itaparica de Carvalho Santiago, Ana Paula França, Carla Magda Allan Santos Domingues, José Cássio de Moraes, Maria da Gloria Teixeira, ICV 2020 Group, Adriana Ilha da Silva, Alberto Novaes Ramos, Ana Paula França, Andrea de Nazaré Marvão Oliveira, Antonio Fernando Boing, Carla Magda Allan Santos Domingues, Consuelo Silva de Oliveira, Ethel Leonor Noia Maciel, Ione Aquemi Guibu, Isabelle Ribeiro Barbosa Mirabal, Jaqueline Caracas Barbosa, Jaqueline Costa Lima, José Cássio de Moraes, Karin Regina Luhm, Karlla Antonieta Amorim Caetano, Luisa Helena de Oliveira Lima, Maria Bernadete de Cerqueira Antunes, Maria da Gloria Teixeira, Maria Denise de Castro Teixeira, Maria Fernanda de Sousa Oliveira Borges, Rejane Christine de Sousa Queiroz, Ricardo Queiroz Gurgel, Rita Barradas Barata, Roberta Nogueira Calandrini de Azevedo, Sandra Maria do Valle Leone de Oliveira, Sheila Araújo Teles, Silvana Granado Nogueira da Gama, Sotero Serrate Mengue, Taynãna César Simões, Valdir Nascimento, Wildo Navegantes de Araújo

**Affiliations:** 1 Universidade Federal do Ceará, Faculdade de Medicina, Fortaleza, CE, Brazil; 2 Universidade Federal da Bahia, Instituto de Saúde Coletiva, Salvador, BA, Brazil; 3 Universidade de Pernambuco, Faculdade de Ciências Médicas, Recife, PE, Brazil; 4 Universidade Federal do Piauí, Campus Senador Helvídio Nunes de Barros, Picos, PI, Brazil; 5 Universidade Federal do Maranhão, Departamento de Saúde Pública, São Luís, MA, Brazil; 6 Faculdade de Ciências Médicas da Santa Casa de São Paulo, São Paulo, SP, Brazil; 7 Organização Pan-Americana da Saúde, Brasília, DF, Brazil; Universidade Federal do Espírito Santo, Vitória, ES, Brazil; Universidade Federal do Ceará, Departamento de Saúde Comunitária, Fortaleza, CE, Brazil; Faculdade Ciências Médicas Santa Casa de São Paulo, São Paulo, SP, Brazil; Secretaria de Estado da Saúde do Amapá, Macapá, AP, Brazil; Universidade Federal de Santa Catarina, SC, Brazil; Organização Pan-Americana da Saúde, Brasília, DF, Brazil; Instituto Evandro Chagas, Belém, PA, Brazil; Faculdade de Ciências Médicas Santa Casa de São Paulo, Departamento de Saúde Coletiva, São Paulo, SP, Brazil; Universidade Federal de Mato Grosso, Cuiabá, MT, Brazil; Universidade Federal do Paraná, Curitiba, PR, Brazil; Universidade Federal de Goiás, Goiânia, GO, Brazil; Universidade Federal do Piauí, Teresina, PI, Brazil; Universidade de Pernambuco, Faculdade de Ciências Médicas, Recife, PE, Brazil; Instituto de Saúde Coletiva, Universidade Federal da Bahia, Salvador, BA, Brazil; Secretaria de Estado da Saúde de Alagoas, Maceió, AL, Brazil; Universidade Federal do Acre, Rio Branco, AC, Brazil; Universidade Federal do Maranhão, Departamento de Saúde Pública, São Luís, MA, Brazil; Universidade Federal de Sergipe, Aracaju, SE, Brazil; Secretaria Municipal de Saúde, Boa Vista, RR, Brazil; Fundação Oswaldo Cruz, Mato Grosso do Sul, Campo Grande, MS, Brazil; Fundação Oswaldo Cruz, Escola Nacional de Saúde Pública Sergio Arouca, Rio de Janeiro, RJ, Brazil; Universidade Federal do Rio Grande do Sul, Porto Alegre, RS, Brazil; Fundação Oswaldo Cruz, Instituto de Pesquisa René Rachou, Belo Horizonte, MG, Brazil; Secretaria de Desenvolvimento Ambiental de Rondônia, Porto Velho, RO, Brazil; Universidade de Brasília, Brasília, DF, Brazil

**Keywords:** Cobertura de Vacunación, Vacilación a la Vacunación, Vacunación Masiva, Programas de Inmunización, Encuesta Epidemiológica, Vaccination Coverage, Vaccine Hesitancy, Mass Vaccination, Immunization Programs, Epidemiological Surveys

## Abstract

**Objective:**

To analyze vaccination coverage and factors associated with incomplete vaccination in inland municipalities of Northeastern Brazil.

**Methods:**

This was a household survey using cluster sampling conducted in Vitória da Conquista, Bahia state, Caruaru, Pernambuco state, Sobral, Ceará state and Imperatriz, Maranhão state between 2020 and 2022. Vaccination coverage by valid doses and vaccine hesitancy were analyzed, with the odds ratio (OR) estimated and adjusted using logistic regression.

**Results:**

Among 1,847 children, complete vaccination coverage was 49.2% (95%CI 43.9;54.5). Factors associated with incomplete vaccination included: higher income (OR 1.53; 95%CI 1.02;2.31), residence in Sobral (OR 4.35; 95%CI 3.04; 6.21) and >1 child (OR 1.20; 95%CI 1.11;1.32). Parental decision not to vaccinate and difficulties in traveling to vaccination centers contributed to vaccine hesitancy.

**Conclusion:**

Low vaccination coverage and incomplete vaccination were associated with social issues in the socioeconomic strata analyzed.

## Study contributions

**Main results:**

Vaccination coverage of nearly 50%, with incomplete vaccination associated with factors across different social strata, particularly higher income. Vaccine hesitancy is marked by parental decision not to vaccinate and access limitations.

**Implications for services:**

Expanding vaccination coverage should be a priority goal within the healthcare and surveillance network of the SUS in inland municipalities of the Northeast region. Incomplete vaccination requires

**Perspectives:**

The actions of the National Immunization Program need to be strengthened, with context-specific interventions in local territories through microplanning to increase vaccination coverage and reduce vaccine hesitancy.

## RESUMEN

**Objetivo:**

Analizar la cobertura de vacunación y factores asociados a vacunación incompleta en municipios del interior del Nordeste de Brasil.

**Método:**

Se trata de una encuesta domiciliaria con muestreo por conglomerados realizada en Vitória da Conquista, Bahía, Caruaru, Pernambuco, Sobral, Ceará e Imperatriz, Maranhão entre 2020 y 2022. Se analizaron cobertura de vacunación por dosis válidas y la indecisión vacunal, con estimación de odds ratio (OR) ajustada por regresión logística.

**Resultados:**

En 1.847 niños, la cobertura de vacunación completa fue del 49,2% (IC_95_% 43,9;54,5). Los factores asociados con vacunación incompleta incluyeron: mayores ingresos (OR 1,53; IC_95_% 1,02;2,31), vivir en Sobral (OR 4,35; IC _95_% 3,04;6,21) y >1 hijo (OR 1,20; IC_95_% 1,11;1,32). La decisión de padres de no vacunar y dificultades para desplazarse a unidades de vacunación demarcaron indecisión vacunal.

**Conclusión:**

Hubo baja cobertura de vacunación y vacunación incompleta asociada a problemas sociales en los estratos socioeconómicos analizados.

## INTRODUCTION

The positive effect of universal adoption of vaccines in national health systems to ensure the quality of life of populations, in different stages of life, has been evident over time.^1,2^ Internationally recognized, Brazil reached the milestone of 50 years of the National Immunization Program (*Programa Nacional de Imunizações* - PNI) in 2023. Its implementation has been a fundamental and successful strategy within the Brazilian National Health System (*Sistema Único de Saúde* - SUS) for achieving operational and epidemiological targets in controlling vaccine-preventable diseases, particularly through primary health care (PHC) networks.^3-6^

A challenge for the SUS has been the monitoring and evaluation of PNI actions to ensure and sustain its effectiveness given potential epidemiological, operational, political-institutional and socioeconomic changes in the health care network.^1,2,6,7^

Since 2016, the declining trend in vaccination coverage for various vaccine-preventable diseases has been concerned public health authorities.^3^ In 2018, measles reemerged, with cases occurring in the Northeast region.^4^

The increase in poverty and extreme poverty in the country has created vulnerable contexts, increasing health risks,^8,9^ making it necessary to recognize and understand the factors that influence the decreasing estimates of vaccination coverage. in Brazil. The central issue is to reduce the risk of accumulation of susceptible individuals, which could lead to the reemergence and sustained circulation of vaccine-preventable infectious agents in the Brazilian population.^1,10,11^

Vaccine hesitancy is defined as the delay in accepting recommended vaccines or refusal of vaccines despite their availability in health services,^12^ which may be related to the declining vaccination coverage.^1^ This phenomenon is complex, and has been identified as one of the top ten global health threats by the World Health Organization. When strengthened, it could further impact low- and middle-income countries, highlighting the need for investigation within the Brazilian context.^13^

Household surveys have been a valuable strategy for deepening the contextual analysis of the varying degrees of achievement of the PNI goals, expanding the evidence in real contexts of the SUS.^10^ In order to broaden knowledge about vaccination coverage in Brazil, this study aims to analyze this coverage and the factors associated with incomplete vaccination in a cohort of children born in 2017 and 2018 in inland municipalities of Northeastern Brazil.

## METHODS

### Study design

This was a population-based survey based on a cohort of live births between 2017 and 2018, with data collected in a previous study.^3^

### Setting

The research was conducted in four municipalities with over 180,000 inhabitants located in inland areas of the Northeast region of Brazil: Vitória da Conquista, Bahia state; Caruaru, Pernambuco state; Sobral, Ceará state and Imperatriz, Maranhão state.

Caruaru was the most populous municipality, with 314,912 inhabitants, 7.7% (24,249) of whom were children aged 0 and 4 years, and it had the highest population density (342.07 inhabitants/km²). Sobral was the least populous municipality, with 188,233 inhabitants, 7.1% (15,063) of whom were children aged 0 and 4 years.^14,15^ Caruaru had the highest number of the family health strategy teams (n=76) and the highest number of vaccination rooms (n=64). Vitória da Conquista had the fewest family health strategy teams. (n=38) and the fewest vaccination rooms (n=33).^14^ Comprehensive PHC coverage was found in Sobral. Vitória da Conquista showed the lowest coverage (63.0%).^4^

### Participants

The study population consisted of children born alive in 2017 and 2018 residing in the municipalities selected for this research and identified through the Live Birth Information System.

Based on the selected live birth cohort, vaccination trajectories of children were analyzed, from birth to 24 months of age. The regions were divided into socioeconomic strata, according to data from the 2010 census on income and education level of the head of the household.^14^

The first step was the spatial organization of four socioeconomic strata into census tracts: A, B, C and D, with stratum A with representing the best income and education conditions and, subsequently, the other strata, with the worst indicators for stratum D.^3^ In the second step, the addresses of the children born alive in the census tracts were georeferenced to form clusters with 56 or more children in each socioeconomic stratum. The third step included locating the expected number of children through random selection, composing the complex cluster sampling process in the socioeconomic strata.^3^ No losses in the sample were reported.

### Variables, data source and measurement

The fieldwork included interviews with parents or guardians, conducted by selected and trained professionals, between September 2020 and March 2022. During home visits, a standardized instrument was applied, including sociodemographic, economic, clinical-epidemiological data, access to health services in the Brazilian National Health System and immunization records, obtained directly through photographs of the vaccination booklets of the selected children.

The analysis was stratified by socioeconomic strata: A (high); B (medium); C (low) and D (very low). The variables with the following characteristics were selected:

- Family nucleus: Beneficiary of the Bolsa Família Program ([PBF]; yes, no), monthly household income (BRL) (≤1,000.00, 1,001.00–3,000.00, 3,001.00–8,000.00, ≥8,001.00; unknown).14
- About the mother: age group (in years, <20, 20–34, ≥35 and unknown), education level (in years of study: 0–8; 9–12; 13–15; ≥16; unknown), paid employment (yes, no), number of children.14
- About the child: child’s sex (male, female), presence of vaccination booklet (yes, no), use of private services (yes, no), and attendance at daycare or school (yes, no)14

For vaccine hesitancy the following variables were considered: ‘absence of childhood vaccination due to adult decision’, ‘difficulty in taking the child to the vaccination center’, ‘failure to vaccinate despite attending the vaccination center’, ‘considers vaccines important for health’, ‘considers vaccines against eradicated diseases unnecessary’ (no, indifferent, yes), ‘considers vaccines important for neighborhood health’ (no, indifferent, yes), ‘fear of severe reactions’ (no, indifferent, yes) and ‘trust in vaccines distributed by the government’ (no, indifferent, yes).^14^

Complete vaccination, with valid doses³ up to 24 months of age, included the routine childhood vaccination schedule.⁴

- At birth: tuberculosis vaccine (bacillus Calmette-Guérin [BCG]) and hepatitis B vaccine.
- DTwP-HepB-Hib vaccine or diphtheria, tetanus, pertussis, *Haemophilus influenzae type b*, hepatitis B; inactivated polio vaccine (IPV); 10-valent pneumococcal conjugate vaccine (PCV10); and rotavirus vaccine.
- 3-5 months: meningococcal C vaccine (MenC).
- 6 months: DTwP-HepB-Hib vaccine or diphtheria, tetanus, pertussis, *Haemophilus influenzae type b*, hepatitis B and IPV.
- 12 months : MMR vaccine or measles, mumps and rubella (MMR), MenC and PCV10 vaccine.
- 15 months: diphtheria, tetanus, and pertussis (DTP) vaccine; hepatitis A vaccine, MMR; oral poliovirus vaccine (OPV); and varicella vaccine.

DTwP-HepB-IPV-Hib vaccines and meningococcal ACWY (MenACWY) vaccine, administered in the private sector, were also included in the analyses. The yellow fever vaccine was not considered in the study, as it was not part of the routine childhood vaccination schedule in some states of the country during the research period.

The proportion of fully vaccinated children (last doses in the vaccination schedule) up to 24 months of age was calculated for the population of live births in 2017 and 2018 in the municipalities studied.

Vaccination coverage progression up to 24 months of age was assessed, following the vaccination schedule and the sequence of doses proposed by the PNI. The evaluation point for each vaccine’s coverage considered the child’s previous doses (cascade vaccination coverage), analyzing the completeness of all doses administered as per the vaccination schedule, by socioeconomic stratum, municipality, and total population.

### Statistical methods

Sample weights for households and each child were calculated based on selection probability, calibrated by population groups, and adjusted for nonresponse and design effect.^3,14^

Weighted estimates of vaccination coverage and respective confidence intervals (95%CI) were calculated for each vaccine and complete vaccination schedule. Considering the complex sampling design, a p-value of <0.05 was used for statistical significance.^3,14^

Risk factors for incomplete vaccination were analyzed by using logistic regression, with adjusted odds ratio (OR) and 95%CI. The analyzed variables with an association of p-value <0.20 in the simple logistic regression analysis, with the calculation of the unadjusted OR, were included in the adjusted model, using the stepwise method. This step investigated the independent effect of these variables, when together, on incomplete vaccination. Collinearity between explanatory variables of the model was assessed using variance inflation factor, excluding the analysis of collinear variables (>20%).

For the dependent variable, vaccination status at 24 months of age, children were dichotomized into ‘fully vaccinated’ (all full scheduled doses received, as the reference group) or ‘not fully vaccinated’ (no doses received or at least one scheduled dose missed), representing incomplete vaccination.

Vaccine hesitancy patterns, i.e. delay in accepting or refusal of recommended vaccines when available in health services, were descriptively analyzed based on the World Health Organization’s “3Cs” model: confidence (knowledge and perceptions of safety and efficacy); convenience (availability, accessibility of vaccination services, access to information and capacity to understand); and complacency (low individual perception of the risk of vaccine-preventable diseases and value attributed to vaccines). ^,6,10^ Parents or guardians of the children in the study were asked whether vaccines were considered important, necessary, reliable, whether they provided collective protection and caused adverse reactions, to characterize attitudes unfavorable, indifferent or favorable to the adoption of the actions proposed in the immunization programs.^3^

It is noted that vaccine hesitancy represents a phenomenon situated between acceptance and total refusal of vaccination, which can vary over time, location and types of vaccines used, emphasizing the importance of vaccination surveys.^13,16^ Stata version 17 (StataCorp LLC, College Station, TX) was used for statistical analysis.

### Ethical aspects

This study approved by the Rsearch Ethics Committees of the Instituto de Saúde Coletiva da Universidade Federal da Bahia, Opinion No. 3,366,818, of June 4, 2019, Certificate of Submission for Ethical Appraisal (CAAE) 4306919.5.0000.5030; and of the Irmandade da Santa Casa de São Paulo, Opinion No. 4,380,019, of November 4, 2020, CAAE 39412020.0.0000.5479.

## RESULTS

Based on a cohort of 40,242 newborns, the study population was selected, consisting of 1,847 children, proportionally distributed across strata and municipalities (Table 1).

**Table 1:** Sociodemographic characteristics of the family, mother and children born between 2017 and 2018 in Caruaru, Imperatriz, Sobral and Vitória da Conquista, according to socioeconomic stratum and 95% confidence interval (95%CI), Brazil, 2020-2022 (n=1,847)

Variable	A (%)	B (%)	C (%)	D (%)	Total (%)
**Municipalities**					
Caruaru	113 (25.0)	114 (24.8)	116 (24.8)	119 (25.4)	462 (25.0)
Imperatriz	120 (26.5)	113 (24.6)	118 (25.2)	114 (24.4)	465 (25.2)
Sobral	103 (22.8)	119 (25.9)	120 (25.6)	123 (26.3)	465 (25.2)
Vitória da Conquista	116 (25.7)	113 (24.6)	114 (24.4)	112 (23.9)	455 (24.6)
Total	452 (24.5)	459 (24.9)	468 (25.3)	468 (25.3)	1,847 (100.0)
**Family characteristics**					
**Programa Bolsa Família**	**%(95%CI)**	**%(95%CI)**	**%(95%CI)**	**%(95%CI)**	**%(95%CI )**
Yes	23.9 (15.6;34.9)	32.9 (23.6;43.7)	44.4 (35.9;53.2)	50.0 (42.5;57.4)	42.5 (37.8;47.3)
**Monthly household income (BRL)**					
≤ 1,000.00	14.3 (7.2;26.4)	28.2 (19.8;38.5)	37.4 (24.0 ; 53.0 )	56.4 (44.0 ; 68.1)	41.5 (34.2;49.3)
1,001.00-3,000.00	29.7 (19.4;42.4)	44.3 (36.3;52.6)	50.2 (36.9;63.5)	34.5 (24.8;45.7)	39.9 (33.6;46.6)
3,001.00-8,000.00	29.2 (20.4;39.9)	16.9 (10.3;26.5)	10.5 (5.6;19.0)	3.2 (1.5;6.9)	10.6 (7.9;14)
≥ 8,001.00	4.8 (2.3;9.9)	1.5 (0.6;4.0)	0.9 (0.3;2.6)	0.0 (0.0;0.0)	1.1 (0.6;1.8)
Unknown	22.1 (12.0;37.1)	9.1 (4.6;17.0)	1.0 (0.5;2.4)	5.9 (2.3;14.2)	7.0 (4.5;10.7)
**Maternal characteristics**					
**Age group (years)**					
<20	1.6 (0.6;4.5)	0.7 (0.2;2.7)	2.0 (1.0;3.8)	1.5 (0.6;3.5)	1.5 (0.9;2.4)
20-34	66.7 (54.2;77.2)	59.3 (47.0;70.6)	70.4 (60.1;79.0)	65.5 (60.4;70.3)	66.0 (61.5 ;70.2)
≥35	31.2 (21;43.6)	39.4 (28.1;51.9)	27.4 (18.7;38.3)	31.1 (25.8;37)	31.5 (27.1;36.2)
**Education level (years)**					
0-8	9.0 (4.8;16.5)	14.9 (8.6;24.5)	6.0 (3.5;9.9)	19.7 (13.4;27.9)	13.8 (10.7;17.7)
9-12	5.7 (3.5;9.1)	15.4 (10.2;22.5)	22.6 (14.8;33.0)	28.5 (18.9;40.5)	22.0 (16.7;28.4)
13-15	40.9 (30.2;52.5)	46.5 (35.9;57.4)	60.9 (50.3;70.6)	41.5 (33.6;49.9)	47.7 (42.3;53.0)
≥16	33.1 (24.8;42.7)	21.4 (14.9;29.8)	9.0 (5.6 ;14.2)	7.7 (4.4;13.2)	13.3 (10.6;16.5)
Unknown	11.3 (4.5;25.7)	1.9 (0.8;4.3)	1.5 (0.5;4.8)	2.6 (1.1;5.9)	3.2 (1.9;5.3)
**Paid employment**	54.7 (42.3;66.6)	50.8 (39.5;62.1)	46.6 (37.7;55.8)	40.7 (33.6;48.2)	45.7 (40.7;50.7)
**Average number of living children per mother**	2.08 (1.98;2.19)	2.13 (2.02;2.24)	2.15 (2.04;2.25)	2.25 (2.14;2.37)	2.15 (2.10;2.21)
**Child characteristics**					
**Sex**					
Masculine	59.9 (51.3;68.0)	51.3 (45.9;56.7)	48.8 (43.8;53.7)	50.9 (44.5;57.3)	51.4 (48.0 ; 54.9)
Feminine	40.1 (32.0;48.7)	48.7 (43.3;54.1)	51.2 (46.3;56.2)	49.1 (42.7;55.6)	48.6 (45.1;52.1)
**Possession of vaccination booklet**	98.2 (92.8;99.6)	99.0 (97.1;99.6)	98.5 (96.6;99.3)	98.5 (92.0;99.8)	98.6 (96.7;99.4)
**Use of private service for vaccination**	13.2 (8.2;20.6)	9.0 (3.8;19.9)	7.5 (4.3;12.8)	2.5 (1.1;5.8)	6.2 (4.3;8.9)
**Attends daycare/school**	41.6 (32.0;51.9)	38.6 (27.8;50.6)	43.6 (30.4;57.7)	59.8 (47.8;70.6)	49.6 (42.3;56.9 )

Vaccination records were found for 98.6% (95%CI 96.7;99.4) of the sample, with no difference between strata and municipalities. The use of private vaccination services occurred in 6.2% (95%CI 4.3;8.9) of the children, with the highest proportion in stratum A (13.2%, 95%CI 8.2;20.6) and the lowest in stratum D (2.5%, 95% CI 1.1;5.8) (Table 1).

Less than half of the children were PBF beneficiaries (42.5%; 95%CI 37.8;47.3), more frequent in stratum D (50.0%, 95%CI 42.5;57.4). Family income was equal to or less than BRL 1,000.00 in 41.5% (95%CI 34.2;49.3), with 56.4% (95%CI 44.0;68.1) in stratum D and 14.3% (95%CI 7.2;26.4) in stratum A. Mothers aged 20-34 years represented 66.0% (95%CI 61.5;70.2), with the highest prevalence in stratum C (70.4%, 95%CI 60.1;79.0) and stratum B (59.3%, 95%CI 47.0;70.6). Regarding education level, 47.7% (95%CI 42.3;53.0) reported having 13-15 years of study, with differences between strata (60.9% [95%CI 50.3;70.6] in stratum C and 40.9% [ 95%CI 30.2;52.5] in stratum A). Paid employment was reported by 45.7% (95%CI 40.7;50.7) of parents or guardians, ranging from 40.7% (95%CI 33.6;48.2) in stratum D to 54.7% (95%CI 42.3;66.6) in stratum A. The average number of children per mother was 2.15 (95%CI 2.10;2.21). The most frequent characteristics were: male gender (51.4%, 95%CI 48.0;54.9) and attending daycare/school (49.6%, 95%CI 42.3;56.9) (Table 1).

**Figure 1:**
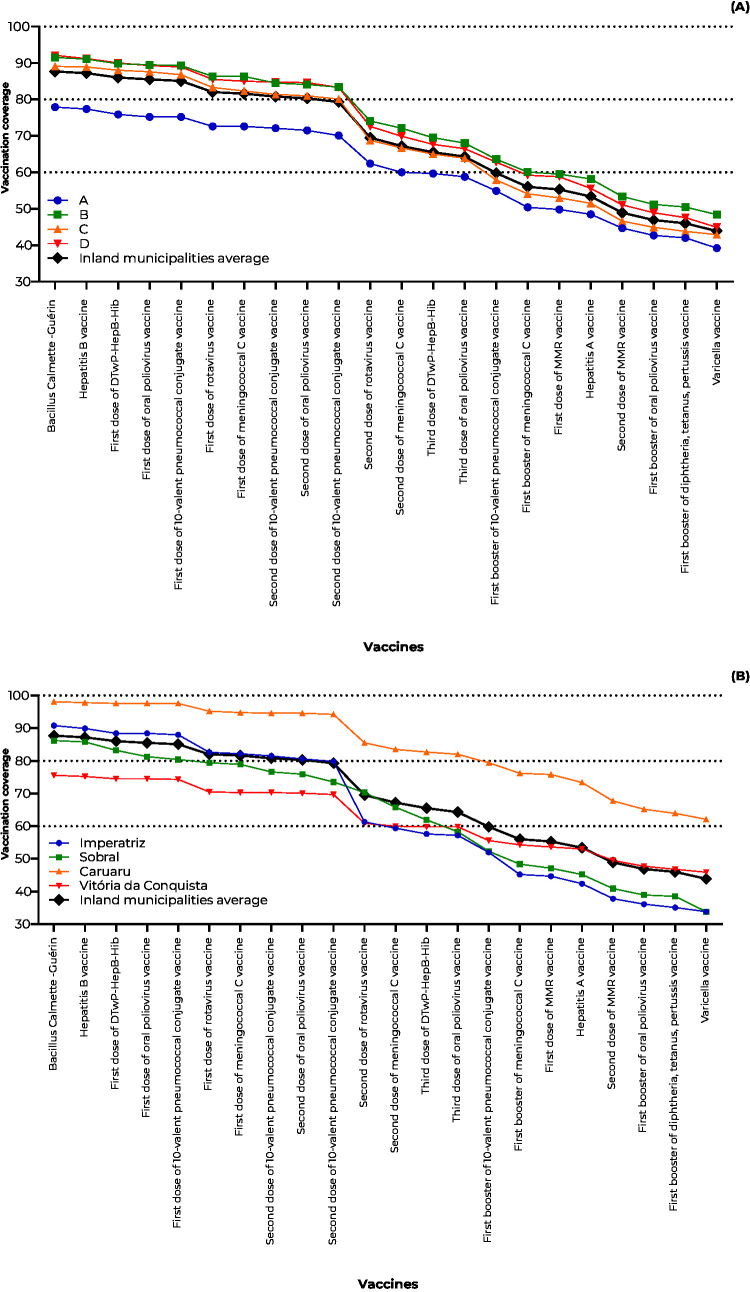
Evolution of vaccine coverage cascade in valid doses, in children born between 2017 and 2018 in Caruaru, Imperatriz, Sobral and Vitória da Conquista, according to socioeconomic stratum (A) and municipality of residence (B), 2020-2022 (n=1,847)

There was a reduction in vaccination coverage within the first 24 months of life across all municipalities and strata. The lowest vaccination coverage of the complete schedule was in stratum A (BCG at birth: 77.9%; 24 months: varicella vaccine: 39.2%), and the highest was in stratum B (BCG at birth: 91.5%; 24 months: varicella vaccine: 48.4%) (Figure 1A). Imperatriz (BCG at birth: 90.8%; 24 months: varicella vaccine: 33.8%) and Sobral (BCG at birth: 33.8%) showed the lowest follow-up vaccination coverage rates, which were lower than the consolidated rates across the municipalities studied (at birth BCG: 87.7%; 24 months: varicella vaccine: 43.9%) (Figure 1B).

**Figure 2:**
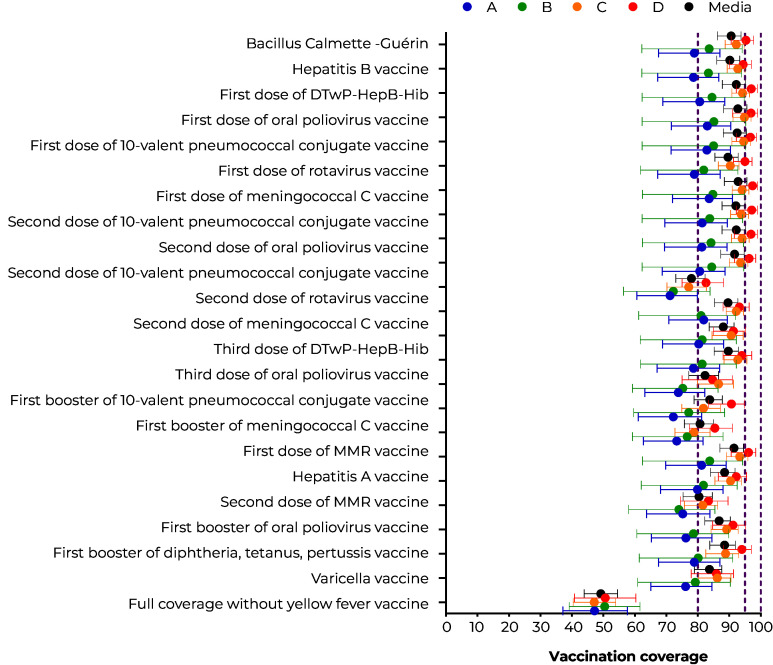
Vaccination coverage by valid doses of immunizers, according to socioeconomic strata, in children born alive in 2017 and 2018, residing in Caruaru, Imperatriz, Sobral and Vitória da Conquista (n = 1,847)

Complete vaccination coverage was 49.2% (95%CI 43.9;54.5), with the highest proportion in stratum D (50.6%, 95%CI 40.8;60.3) and in Caruaru (57.7%, 95%CI 49.2;65.7) and the lowest in stratum C (47.1%, 95%CI 40.4;53.9) and in Imperatriz (34.2%, 95%CI 28.7;40.1). The highest coverage (92.8%, 95%CI 88.4;95.5) was observed for the first dose of the MenC vaccine. The lowest coverage was for OPV versus the second dose of the rotavirus vaccine (78.0%, 95%CI 73.0;82.3) (Figure 2).

The outcome was more significantly observed in children residing in Sobral (OR 4.35; 95%CI 3.04;6.21), with income between BRL 3,001.00-R$8,000.00 (adjusted OR1.53; 95%CI 1.02;2.31) and mothers with more than 1 child (OR 1.20; 95%CI 1.11;1.32) (Table 2).

**Table 2:** Crude and adjusted odds ratios (OR) and 95% confidence intervals (95%CI) for incomplete vaccination in children born in 2017 and 2018, according to family, maternal and child characteristics. Caruaru, Imperatriz, Sobral and Vitória da Conquista, Brazil, 2020-2022 (n = 1,847)

Variables	Crude OR (95%CI)	p-value	Adjusted OR (95%CI)	p-value
Socioeconomic stratum		0.877		-
A	1.44 (1.11 ;1.87)		-	
B	1.00		-	
C	1.19 (0.92 ; 1.54)		-	
D	1.12 (0.87 ; 1.45)		-	
**Municipalities**		<0.001		0.005
Caruaru	1.00		1.00	
Imperatriz	3.07 (2.35;4.01)		2.80 (1.93 ; 3.80)	
Sobral	3.15 (2.41;4.13)		4.35 (3.04 ; 6.21)	
Vitória da Conquista	1.90 (1.46;2.47)		1.63 (1.19 ; 2.23)	
**Family characteristics**				
**Programa Bolsa Família**		0.699		-
Yes	1.00		-	
No	1.04 (0.86 ; 1.25)		-	
**Monthly household income (in BRL)**		<0.001		0.001
≤1,000.00	1.00		1.00	
1,001.00-3,000.00	1.27 (1.03;1.56)		0.98 (0.75;1.28)	
3,001.00-8,000.00	1.96 (1.44;2.65)		1.53 (1.02;2.31)	
≥8,001.00	2.79 (1.46;5.36)		2.06 (0.94;4.48)	
**Maternal characteristics**				
**Age at child’s birth (years)**		0.869		-
<20	1.00		-	
20-34	0.78 (0.37 ; 1.61)		-	
≥35	0.78 (0.37 ; 1.64)		-	
**Education level (years)**		0.006		0.907
0-8	1.00		1.00	
9-12	1.03 (0.75 ; 1.41)		0.92 (0.65;1.31)	
13-15	1.11 (0.84 ; 1.45)		0.77 (0.55;1.08)	
≥16	1.63 (1.17;2.28)		0.89 (0.57;1.41)	
**Paid employment**		0.004		0.330
Yes	1.00		1.00	
No	1.31 (1.09;1.58)		1.25 (0.99;1.58)	
**Living children by mother**		<0.001		<0.001
Average	1.17 (1.08;1.26)		1.20 (1.11 ; 1.32)	
Child characteristics				
**Sex**		0.479		-
Masculine	1.00		-	
Feminine	1.07 (0.89 ; 1.28)		-	
**Possession of vaccination booklet**		0.181		0.261
Yes	1.00		1.00	
No	2.45 (0.66 ; 9.06)		0.47 (0.12;1.83)	
**Use of private service for vaccination**		0.009		0.062
Yes	1.66 (1.14;2.43)		1.33 (0.84;2.12)	
No	1.00		1.00	
**Attends daycare/school**		<0.001		<0.001
Yes	1.72 (1.43;2.07)		1.14 (0.90;1.45)	
No	1.00		1.00	

Mothers or guardians decided not to vaccinate their children in 1.2% of cases, due to the difficulties in taking the child to the vaccination center (8.9%). Despite having been to the health center, 37.1% of children missed at least one vaccination opportunity (Table 3).

**Table 3:** Perception of parents or guardians regarding vaccination, according to socioeconomic stratum, of children born between 2017-2018 in Caruaru, Imperatriz, Sobral and Vitória da Conquista, 2020-2022 (n=1,847)

Variables	A (%)	B (%)	W (%)	D (%)	**Total (%)**
**Absence of childhood vaccination due to adult decision**	3 (0.7)	4 (0.9)	8 (1.7)	8 (1.7)	23 (1,2)
**Difficulty in taking the child to the vaccination center**	47 (10.4)	37 (8.1)	35 (7.5)	46 (9.8)	165 (8.9)
**Absence of vaccination even after taking the child to the vaccination center**	180 (39.8)	161 (35.1)	165 (35.3)	179 (38.2)	685 (37.1)
**Considers vaccines important for health**					
No	1 (0.2)	-	-	2 (0.4)	3 (0.2)
Indifferent	3 (0.7)	1 (0.2)	3 (0.6)	2 (0.4)	9 (0.5)
Yes	448 (99.1)	458 (99.8)	465 (99.4)	464 (99.1)	1,835 (99.4)
**Considers vaccines against eradicated diseases unnecessary**					
Yes	53 (11.7)	32 (7.0)	81 (17.3)	69 (14.7)	235 (12.7)
Indifferent	29 (6.4)	32 (7.0)	58 (12.4)	17 (3.6)	136 (7.4)
No	370 (81.9)	395 (86.1)	329 (70.3)	382 (81.6)	1,476 (79.9)
**Considers vaccines important for neighborhood health**					
No	2 (0.4)	-	1 (0.2)	5 (1.1)	8 (0.4)
Indifferent	24 (5.3)	26 (5.7)	23 (4.9)	5 (1.1)	78 (4.2)
Yes	426 (94.2)	433 (94.3)	444 (94.9)	458 (97.9)	1,761 (95.3)
**Fear of severe reactions**					
Yes	95 (21.0)	93 (20.3)	87 (18.6)	92 (19.7)	367 (19.9)
Indifferent	42 (9.3)	26 (5.7)	65 (13.9)	36 (7.7)	169 (9.1)
No	315 (69.7)	340 (74.1)	316 (67.5)	340 (72.6)	1,311 (71.0)
**Trust in vaccines provided by the government**					
No	7 (1.5)	6 (1.3)	6 (1.3)	5 (1.1)	24 (1.3)
Indifferent	12 (2.7)	11 (2.4)	11 (2.4)	15 (3.2)	49 (2.7)
Yes	433 (95.8)	442 (96.3)	451 (96.4)	448 (95.7)	1,774 (96.0)

Vaccines were considered important health interventions by 99.4% of mothers or guardians. In the study population, there were reports that they ‘*considered vaccines against eradicated diseases unnecessary*’ (12.7%), ‘*considered vaccines important for the* neighborhood health’ (0.4%), ‘*feared severe side effects* ‘ (19.9%) and ‘ *did not trust the vaccines provided by the government*’ (1.3%) (Table 3).

## DISCUSSION

Low vaccination coverage was observed among children up to two years of age living in the four inland municipalities investigated of the Northeastern Brazil. Complete vaccination coverage in Imperatriz was found in one-third of the children, and in Caruaru, just over half of the children showed a complete vaccination schedule. A significant portion of children were thus susceptible to vaccine-preventable diseases in the municipalities studied.

None of the immunobiological agents evaluated reached the coverage target recommended by the PNI, with heterogeneous rates observed, notably lower for multidose vaccines. The few exceptions occurred with vaccines administered at the beginning of the routine childhood vaccination, especially in the lowest socioeconomic stratum, which achieved higher vaccination coverage for vaccines administered at birth. The main causes of vaccine hesitancy reported reflect issues related to parental decision against vaccination, access restriction due to transportation difficulties, and operational aspects within vaccination units.

Low vaccination coverage rates suggest that inland municipalities of the Northeast region contribute to the decline and heterogeneity that have been observed in the country since 2016,^4,6^ including high dropout rates, as highlighted in the results showing a cascade of diminishing vaccination coverage. The complexity of the childhood vaccination schedule with the introduction of new vaccines, barriers to access due to transport difficulties and opening hours of vaccination rooms, insufficient general logistics and infrastructure, occasional shortage of immunobiological agents and supplies in PHC, vaccine hesitancy, misinformation about vaccines and sociocultural determinants were the main causes reported by the respondents in this survey. These factors align with those cited in both national and international literature.^1,6,17-19^

It is worth noting that missed opportunities for vaccination occurred across all socioeconomic strata, especially in the highest one. This indicates the need to develop strategies aimed at different socioeconomic contexts in order to overcome this issue, and qualitative research is crucial to better understand the underlying determinants.

The association between incomplete vaccination and mothers without paid employment and with more than one child was similar to that observed in other studies conducted in Brazil in previous decades.^10,11^ This suggests that social vulnerability may be one of the factors that contributes to incomplete vaccination and non-compliance with the vaccination schedule, a pattern already observed in other studies in Brazil and in low-income countries.^11,20,21^

Conditional cash transfer policies tied to childhood vaccination have proven effective. ^22,23^ This was evident in this study, which revealed higher vaccination coverage in the lowest socioeconomic stratum, which accounts for the highest proportion of PBF beneficiaries. In stratum C, vaccination coverage was below the desired level. This highlights the existence of greater vaccine hesitancy among disadvantaged populations who are not encouraged to comply with the PBF condition and who, due to their low level of education, may experience greater uncertainty and ambiguity regarding vaccine information.^24^

Although a low proportion of parents or guardians used private vaccination services, this variable was associated with incomplete vaccination, probably due to limited information on follow-up data for children vaccinated in public services, as well as for children vaccinated in private services, where vaccine dose records are not systematically shared with public health surveillance.^11^ Enhancing the follow-up of children vaccinated in private services should be considered in strategies to restore coverage to pre-2016 levels. This underscores the need for ongoing dialogue with private service managers to facilitate data sharing with public services as part of the national effort to protect the entire child population.^25^

The success achieved by the PNI – through the continuous implementation of health education, communication and information strategies together with society to control and eliminate vaccine-preventable diseases, as well as an effective and widespread structure of vaccination actions – have not been maintained. The rise of misinformation, especially related to COVID-19, strengthened the growing vaccine denial movement in recent years,^1,^ ^26^ has reduced the population’s risk perception regarding the re-emergence of vaccine-preventable diseases and may have hindered the achievement of desirable vaccination coverage for the country, including inland municipalities of the Northeast region.

Initiatives have been adopted by the Ministry of Health, aimed at strengthening the PNI^4^ and in collaboration with the PHC, including expanded educational campaigns, widen vaccination rooms and extend operating hours in the regions, considering their specificities. These initiatives represent efforts to ensure the sustainability of vaccination actions within the Brazilian National Health System, aimed at achieving further advances in improving the health status of the Brazilian population.

This study has limitations, particularly due to the issues regarding the absence of the 2020 census, which required the use of data from 2010. This study may present discrepancies for some areas compared to current data, used for socioeconomic strata definition. In addition, there are inherent challenges in household surveys, such as distrust, insecurity or lack of interest in participating, especially in higher-income strata, which were exacerbated by the need for social distancing due to the COVID -19 pandemic. This issue was mitigated by increasing the number of children selected in each stratum and extending the data collection period. Furthermore, there were difficulties in reading vaccination booklets, due to non-standardized records, poor legibility or recording errors. To address these issues, the reading was conducted by trained professionals and, when necessary, consultations with the PNI Information System were performed.

Operational issues and misinformation were highlighted. Despite all these challenges, awareness of the importance of vaccination was found among the study population.

The results presented in this study, in addition to others derived from the national survey, provide essential and strategic information for public health management, especially for macroplanning.^27^ Local strategies that are cost-effective and participatory should be developed to implement appropriate and effective vaccination activities and achieve the targets associated with these strategic indicators, which directly reflect the health conditions of the population, particularly children.

In conclusion, low vaccination coverage and incomplete vaccination were observed in children particularly in Sobral, but also in Caruaru, Imperatriz and Vitória da Conquista. People with higher income and more than one child showed a higher risk for incomplete vaccination. The use of private vaccination services, together with the absence of paid employment and low education level, may also indicate contexts of vulnerability. Parents’ or guardians’ decision not to vaccinate and the limited access to health care due to difficulties in traveling to vaccination centers were relevant factors linked to vaccine hesitancy in the cohort analyzed. This context includes critical operational aspects of the vaccination rooms within the SUS, with significant implications for unvaccinated children, even when present at vaccination units.
