# Supplementary material for: Vaccination coverage, vaccine hesitancy and factors associated with incomplete vaccination: a household survey conducted with children born between 2017 and 2018 in the inland municipalities of Northeastern Brazil
Source: Epidemiol Serv Saude. 2025 Jan 10;33(spe2):e20231224. doi: 10.1590/S2237-96222024v33e20231224.especial2.en (PMC11734601; doi:10.1590/S2237-96222024v33e20231224.especial2.en)
Supplement: Translation

# Translation: Cobertura, hesitação vacinal e fatores associados à vacinação incompleta: inquérito domiciliar com crianças nascidas no período 2017-2018 em municípios do interior do Nordeste brasileiro

**DOI:** 10.1590/S2237-96222024v33e20231224.especial2.pt

**Authors:** Anderson Fuentes Ferreira, Alberto Novaes Ramos, Adjoane Mauricio Silva Maciel, Jaqueline Caracas Barbosa, Ramon da Costa Saavedra, Maria Bernadete de Cerqueira Antunes, Luisa Helena de Oliveira Lima, Rejane Christine de Sousa Queiroz, Taynara Lais Silva, Martha Suely Itaparica de Carvalho Santiago, Ana Paula França, Carla Magda Allan Santos Domingues, José Cássio de Moraes, Maria da Gloria Teixeira, Grupo ICV 2020, Adriana Ilha da Silva, Alberto Novaes Ramos, Ana Paula França, Andrea de Nazaré Marvão Oliveira, Antonio Fernando Boing, Carla Magda Allan Santos Domingues, Consuelo Silva de Oliveira, Ethel Leonor Noia Maciel, Ione Aquemi Guibu, Isabelle Ribeiro Barbosa Mirabal, Jaqueline Caracas Barbosa, Jaqueline Costa Lima, José Cássio de Moraes, Karin Regina Luhm, Karlla Antonieta Amorim Caetano, Luisa Helena de Oliveira Lima, Maria Bernadete de Cerqueira Antunes, Maria da Gloria Teixeira, Maria Denise de Castro Teixeira, Maria Fernanda de Sousa Oliveira Borges, Rejane Christine de Sousa Queiroz, Ricardo Queiroz Gurgel, Rita Barradas Barata, Roberta Nogueira Calandrini de Azevedo, Sandra Maria do Valle Leone de Oliveira, Sheila Araújo Teles, Silvana Granado Nogueira da Gama, Sotero Serrate Mengue, Taynãna César Simões, Valdir Nascimento, Wildo Navegantes de Araújo

**Affiliations:** 1 Universidade Federal do Ceará, Faculdade de Medicina, Fortaleza, CE, Brasil; 2 Universidade Federal da Bahia, Instituto de Saúde Coletiva, Salvador, BA, Brasil; 3 Universidade de Pernambuco, Faculdade de Ciências Médicas, Recife, PE, Brasil; 4 Universidade Federal do Piauí, Campus Senador Helvídio Nunes de Barros, Picos, PI, Brasil; 5 Universidade Federal do Maranhão, Departamento de Saúde Pública, São Luís, MA, Brasil; 6 Faculdade de Ciências Médicas da Santa Casa de São Paulo, São Paulo, SP, Brasil; 7 Organização Pan-Americana da Saúde, Brasília, DF, Brasil; Universidade Federal do Espírito Santo, Vitória, ES, Brasil; Universidade Federal do Ceará, Departamento de Saúde Comunitária, Fortaleza, CE, Brasil; Faculdade Ciências Médicas Santa Casa de São Paulo, São Paulo, SP, Brasil; Secretaria de Estado da Saúde do Amapá, Macapá, AP, Brasil; Universidade Federal de Santa Catarina, SC, Brasil; Organização Pan-Americana da Saúde, Brasília, DF, Brasil; Instituto Evandro Chagas, Belém, PA, Brasil; Faculdade de Ciências Médicas Santa Casa de São Paulo, Departamento de Saúde Coletiva, São Paulo, SP, Brasil; Universidade Federal de Mato Grosso, Cuiabá, MT, Brasil; Universidade Federal do Paraná, Curitiba, PR, Brasil; Universidade Federal de Goiás, Goiânia, GO, Brasil; Universidade Federal do Piauí, Teresina, PI, Brasil; Universidade de Pernambuco, Faculdade de Ciências Médicas, Recife, PE, Brasil; Instituto de Saúde Coletiva, Universidade Federal da Bahia, Salvador, BA, Brasil; Secretaria de Estado da Saúde de Alagoas, Maceió, AL, Brasil; Universidade Federal do Acre, Rio Branco, AC, Brasil; Universidade Federal do Maranhão, Departamento de Saúde Pública, São Luís, MA, Brasil; Universidade Federal de Sergipe, Aracaju, SE, Brasil; Secretaria Municipal de Saúde, Boa Vista, RR, Brasil; Fundação Oswaldo Cruz, Mato Grosso do Sul, Campo Grande, MS, Brasil; Fundação Oswaldo Cruz, Escola Nacional de Saúde Pública Sergio Arouca, Rio de Janeiro, RJ, Brasil; Universidade Federal do Rio Grande do Sul, Porto Alegre, RS, Brasil; Fundação Oswaldo Cruz, Instituto de Pesquisa René Rachou, Belo Horizonte, MG, Brasil; Secretaria de Desenvolvimento Ambiental de Rondônia, Porto Velho, RO, Brasil; Universidade de Brasília, Brasília, DF, Brasil

**Keywords:** Cobertura Vacinal, Hesitação Vacinal, Vacinação em Massa, Programas de Imunização, Inquéritos Epidemiológicos

## Abstract

**Objetivo:**

Analisar a cobertura vacinal e os fatores associados à vacinação incompleta em municípios do interior do Nordeste brasileiro.

**Métodos:**

Trata-se de inquérito domiciliar com amostragem por conglomerados realizado em Vitória da Conquista – Bahia, Caruaru – Pernambuco, Sobral – Ceará e Imperatriz – Maranhão, entre 2020 e 2022. Analisou-se a cobertura vacinal por doses válidas e a hesitação vacinal, com estimativa da razão de chances (*odds ratio*, OR) ajustada por regressão logística.

**Resultados:**

Em 1.847 crianças, a cobertura vacinal completa foi 49,2% (IC_95_% 43,9;54,5). Entre fatores associados à vacinação incompleta destacam-se: maior renda (OR 1,53; IC_95_% 1,02;2,31), residência em Sobral (OR 4,35; IC_95_% 3,04;6,21) e >1 filho (OR 1,20; IC_95_% 1,11;1,32). Decisão de não vacinação pelos pais e dificuldades no deslocamento às unidades de vacinação demarcaram a hesitação vacinal.

**Conclusão:**

Verificou-se baixas coberturas vacinais e vacinação incompleta associada a questões sociais nos estratos socioeconômicos analisados.

## Contribuições do estudo

**Principais resultados:**

Cobertura vacinal de quase 50%, com vacinação incompleta associada a fatores nos diferentes estratos sociais, particularmente de maior renda. Hesitação vacinal é demarcada por decisão de não vacinação pelos pais e limitações de acesso.

**Implicações para os serviços:**

A ampliação da cobertura vacinal deve ser colocada como meta prioritária na rede de atenção e vigilância do SUS em municípios do interior do Nordeste. A incompletude vacinal requer

**Perspectivas:**

As ações do Programa Nacional de Imunizações precisam ser potencializadas, com ações contextualizadas nos territórios por meio de microplanejamento a fim de aumentar as coberturas vacinais e reduzir a hesitação.

## INTRODUÇÃO

O efeito positivo da adoção universal de vacinas em sistemas nacionais de saúde para a garantia de qualidade de vida das populações, nos diferentes ciclos de vida, tem sido evidente ao longo do tempo.^1,2^ Destaque internacional, o Brasil alcançou em 2023 a marca de 50 anos do Programa Nacional de Imunizações (PNI). Sua implementação tem sido estratégia fundamental e exitosa no Sistema Único de Saúde (SUS) para alcance de parâmetros operacionais e epidemiológicos de controle de doenças imunopreveníveis, particularmente via redes de atenção primária à saúde (APS).^3-6^

Um desafio para o SUS tem sido monitorar e avaliar ações do PNI para garantir e sustentar a sua efetividade diante de eventuais mudanças epidemiológicas, operacionais da rede de atenção à saúde, político-institucionais e socioeconômicas.^1,2,6,7^

Desde 2016, a tendência de redução da cobertura vacinal para diferentes doenças imunopreveníveis tem preocupado autoridades da saúde pública.^3^ Em 2018, verificou-se o reaparecimento do sarampo, com ocorrência de casos na região Nordeste.^4^

O aumento da pobreza e extrema pobreza no país gerou contextos de vulnerabilização, ampliado riscos à saúde,^8,9^ tornando-se necessário reconhecimento e entendimento de fatores que influenciam nas decrescentes estimativas de cobertura vacinal no Brasil. A questão central é reduzir o risco de acúmulo de pessoas suscetíveis capazes de determinar o reaparecimento e a manutenção da circulação de agentes infecciosos imunopreveníveis na população brasileira.^1,10,11^

A hesitação vacinal é definida como retardo em aceitar vacinas recomendadas ou rejeição destas, apesar da disponibilidade de tais vacinas nos serviços de saúde,^12^ que pode estar relacionada aos contextos de declínios de cobertura vacinal.^1^ Esse fenômeno é complexo, considerado como uma das dez maiores ameaças mundiais à saúde pública pela Organização Mundial da Saúde. Quando fortalecido, poderá impactar ainda mais países de média e baixa renda, ressaltando a necessidade de sua investigação no contexto brasileiro.^13^

Inquéritos domiciliares têm sido estratégia relevante para aprofundar a análise contextual dos diferentes graus de alcance das metas do PNI, ampliando as evidências em contextos reais do SUS.^10^ Com a perspectiva de expandir o conhecimento sobre as coberturas vacinais no Brasil, este estudo tem como objetivo analisar essas coberturas e os fatores associados à vacinação incompleta em coorte de crianças nascidas em 2017 e 2018 em municípios do interior do Nordeste brasileiro.

## MÉTODOS

### Delineamento

Trata-se de inquérito de base populacional a partir de coorte de nascidos vivos entre 2017 e 2018, com dados coletados em estudo prévio.^3^

### Contexto

A pesquisa foi desenvolvida em quatro municípios acima de 180 mil habitantes localizados em áreas do interior da região Nordeste do Brasil: Vitória da Conquista – Bahia, Caruaru – Pernambuco, Sobral – Ceará e Imperatriz – Maranhão.

Caruaru foi o município mais populoso, com 314.912 habitantes, dos quais, 7,7% (24.249) eram crianças entre 0 e 4 anos de idade, e com maior densidade demográfica (342,07 habitantes/km²). Sobral foi o município menos populoso, com 188.233 habitantes, com 7,1% (15.063) crianças entre 0 e 4 anos de idade.^14,15^ Caruaru possuía o maior número de equipes vinculadas à estratégia de saúde da família (n=76) e o maior número de salas de vacina (n=64). Vitória da Conquista possuía o menor número de equipes da estratégia de saúde da família (n=38) e o menor número de salas de vacina (n=33).^14^ A cobertura integral da APS foi verificada em Sobral. A menor cobertura foi em Vitória da Conquista (63,0%).^4^

### Participantes

A população deste estudo foi constituída por crianças nascidas vivas em 2017 e 2018 residentes nos municípios selecionados para esta pesquisa e contabilizadas a partir do Sistema de Informação sobre Nascidos Vivos.

A partir da coorte de nascidos vivos selecionada, analisou-se a trajetória de vacinação da criança, desde o nascimento até os 24 meses de idade. Os territórios foram divididos segundo estratos socioeconômicos, compostos a partir de dados oriundos do censo demográfico 2010 sobre renda e escolaridade da pessoa chefe da família.^14^

A primeira etapa consistiu em organizar espacialmente 4 estratos socioeconômicos em setores censitários: A, B, C e D, considerando A, o estrato com melhores condições de renda e de escolaridade e, na sequência, os demais estratos, com piores indicadores para o estrato D.^3^ Na segunda etapa, procedeu-se o georreferenciamento dos endereços de crianças nascidas vivas nos setores censitários para formar conglomerados com 56 ou mais crianças em cada estrato socioeconômico. A terceira etapa incluiu a localização do número previsto de crianças por meio de seleção por sorteio, compondo o processo de amostragem complexa por conglomerados nos estratos socioeconômicos.^3^ Não foram relatadas perdas na amostra.

### Variáveis, fonte de dados e mensuração

O trabalho de campo incluiu entrevistas com pais ou responsáveis, por profissionais selecionados e treinados, entre setembro de 2020 e março de 2022. A partir de visitas domiciliares, aplicou-se instrumento padronizado incluindo dados sociodemográficos, econômicos, clínico-epidemiológicos, de acesso aos serviços de saúde do Sistema Único de Saúde e registro das imunizações, diretamente a partir da fotografia do cartão de vacinação das crianças selecionadas.

A análise foi apresentada segundo estratos socioeconômicos: A (alto); B (médio); C (baixo) e D (muito baixo). Foram selecionadas as variáveis com características:

- Do núcleo familiar: Beneficiário do Programa Bolsa Família ([PBF]; sim, não), renda familiar mensal (R$) (≤1.000,00, 1.001,00–3.000,00, 3.001,00–8.000,00, ≥8.001,00; ignorado).14
- Da mãe: faixa etária (em anos, <20, 20–34, ≥35 e ignorado), escolaridade (em anos de estudo: 0–8; 9–12; 13–15; ≥16; ignorado), trabalho remunerado (sim, não), número de filhos.14
- Da criança: sexo da criança (masculino, feminino), presença de caderneta de vacinação (sim, não), uso de serviço privado (sim, não), e frequência à creche ou escola (sim, não).14

Para a hesitação foram consideradas as variáveis: ‘ausência de vacinação infantil por decisão do adulto’, ‘dificuldade para levar a criança ao posto de vacinação’, ‘ausência de vacinação mesmo ao levar ao posto de vacinação’, ‘considera vacinas importantes para saúde’, ‘considera desnecessárias vacinas contra doenças erradicadas’ (não, indiferente, sim), ‘considera vacinas importantes para saúde do bairro’ (não, indiferente, sim), ‘receio de reações graves’ (não, indiferente, sim) e ‘confiança em vacinas distribuídas pelo governo’ (não, indiferente, sim).^14^

Considerou-se o esquema completo com doses válidas^3^ até os 24 meses de idade, que incluiu o esquema vacinal básico completo.^4^

- Ao nascer: vacina contra tuberculose (bacilo de Calmette-Guérin [BCG]) e vacina contra hepatite B.
- 2-4 meses: vacina pentavalente ou vacina contra difteria, tétano, coqueluche, *Haemophilus influenzae b*, hepatite B; vacina inativada contra poliomielite (VIP); vacina pneumocócica 10-valente (VPC10); e vacina contra rotavírus humano.
- 3-5 meses: vacina meningocócica C (MenC).
- 6 meses: vacina pentavalente ou vacina contra difteria, tétano, coqueluche, *H. influenzae b*, hepatite B e VIP.
- 12 meses: vacina tríplice viral ou vacina contra sarampo, caxumba e rubéola (SCR), MenC e VPC10.
- 15 meses: vacina contra difteria, tétano e pertussis (DTP); vacina contra hepatite A, SCR; vacina oral de poliovírus (VOP); e vacina contra varicela.

As vacinas hexavalente e vacina meningocócica ACWY (MenACWY), aplicadas no setor privado, também foram incluídas nas análises. A vacina contra febre amarela não foi considerada no estudo em virtude de, no período da pesquisa, não compor o esquema vacinal básico em alguns estados do país.

Calculou-se a proporção de crianças completamente vacinadas (últimas doses do esquema vacinal) até os 24 meses de idade na população de nascidos vivos em 2017 e 2018 nos municípios pesquisados. 

Foi avaliada a evolução da cobertura vacinal até os 24 meses de idade, observando o calendário vacinal e a sequência de doses propostas pelo PNI. Considerou-se, como ponto de avaliação para cobertura de cada vacina, o registro de doses anteriores recebidas pela criança (cobertura vacinal em cascata), considerando a completude integral das doses aplicadas, segundo o calendário vacial^12^ caracterizada segundo estrato, município de residência e total.

### Métodos estatísticos

Os pesos da amostra para os domicílios e para cada criança foram calculados com base na probabilidade de seleção, calibrados conforme grupos de populações e ajustados para a não resposta e efeito do projeto.^3,14^

Estimativas ponderadas de cobertura vacinal e respectivos intervalos de confiança (IC_95_%) foram calculados para cada vacina e esquema completo de vacinação. Considerando o complexo desenho amostral, utilizou-se um p-valor<0,05 para significância estatística.^3,14^

Foram analisados os fatores de risco para vacinação incompleta por meio da aplicação de regressão logística, com cálculo da razão de chance (*odds ratio*, OR) ajustada e IC_95_%. As variáveis analisadas que apresentaram associação com p-valor<0,20 na análise por modelo de regressão logística simples, com o cálculo da OR não ajustada, foram incluídas no modelo ajustado, utilizando-se o método *stepwise* para essa seleção. Essa etapa investigou o efeito independente dessas variáveis, quando em conjunto, na vacinação incompleta. A presença de colinearidade entre as variáveis explicativas do modelo foi verificada a partir da análise do fator de inflação da variância, com exclusão da análise de variáveis colineares (>20%).

Para a variável dependente, situação vacinal de vacinação aos 24 meses de idade, procedeu-se à dicotomização em crianças ‘completamente vacinada’ (todas as doses completas previstas, como grupo de referência) ou ‘sem vacinação completa’ (sem nenhuma dose aplicada ou com falta de pelo menos uma dose prevista), representando incompletude vacinal.

Analisaram-se, descritivamente, padrões de hesitação vacinal, ou seja, atraso em aceitar ou recusa em utilizar vacinas recomendadas quando disponíveis nos serviços de saúde, com base no modelo da Organização Mundial da Saúde dos “três Cs”: confiança (conhecimento e percepções da segurança e eficácia); conveniência (disponibilidade, acessibilidade de serviços de vacinação, acesso à informação e capacidade de compreensão); e complacência (baixa percepção individual do risco de doenças imunopreveníveis e valor atribuído a vacinas).^5,6,10^ Procedeu-se à verificação com os pais ou responsáveis pelas crianças do estudo se vacinas são consideradas importantes, necessárias, confiáveis, se mantêm proteção coletiva e produzem reações, para caracterização de dimensões que traduzem aspectos desfavoráveis, indiferentes ou favoráveis à adoção das ações propostas nos programas de imunização.^3^

Registra-se que a hesitação vacinal representa fenômeno situado entre a aceitação e a recusa total da vacinação, podendo variar ao longo do tempo, do local e dos tipos de vacinas utilizadas, daí a importância de inquéritos vacinais.^13,16^ Foi utilizado o *software* Stata versão 17 para os cálculos estatísticos (StataCorp LLC, College Station, TX).

### Aspectos éticos

Pesquisa aprovada pelos comitês de ética em pesquisa com seres humanos do Instituto de Saúde Coletiva da Universidade Federal da Bahia, sob Parecer nº 3.366.818, de 4 de junho de 2019, Certificado de Apresentação de Apreciação Ética (CAAE) 4306919.5.0000.5030; e da Irmandade da Santa Casa de São Paulo, sob Parecer nº 4.380.019, de 4 de novembro de 2020, CAAE 39412020.0.0000.5479.

## RESULTADOS

A partir de 40.242 recém-nascidos, selecionou-se a população do estudo, composta por 1.847 crianças, distribuídas proporcionalmente entre estratos e municípios (Tabela 1).

**Tabela 1:** Características sociodemográficas da família, da mãe e das crianças nascidas entre 2017 e 2018 em Caruaru, Imperatriz, Sobral e Vitória da Conquista, segundo estrato socioeconômico e intervalo de confiança de 95% (IC95%), 2020-2022 (n=1.847)

Variável	A (%)	B (%)	C (%)	D (%)	Total (%)
**Municípios**					
Caruaru	113 (25,0)	114 (24,8)	116 (24,8)	119 (25,4)	462 (25,0)
Imperatriz	120 (26,5)	113 (24,6)	118 (25,2)	114 (24,4)	465 (25,2)
Sobral	103 (22,8)	119 (25,9)	120 (25,6)	123 (26,3)	465 (25,2)
Vitória da Conquista	116 (25,7)	113 (24,6)	114 (24,4)	112 (23,9)	455 (24,6)
Total	452 (24,5)	459 (24,9)	468 (25,3)	468 (25,3)	1.847 (100,0)
**Características da família**					
**Programa Bolsa Família**	**% (IC**_95_ **%** **)**	**% (IC**_95_ **%** **)**	**% (IC**_95_ **%** **)**	**% (IC**_95_ **%** **)**	**% (IC**_95_ **%** **)**
Sim	23,9 (15,6;34,9)	32,9 (23,6;43,7)	44,4 (35,9;53,2)	50,0 (42,5;57,4)	42,5 (37,8;47,3)
**Renda familiar mensal (R$)**					
≤1.000,00	14,3 (7,2;26,4)	28,2 (19,8;38,5)	37,4 (24,0;53,0)	56,4 (44,0;68,1)	41,5 (34,2;49,3)
1.001,00-3.000,00	29,7 (19,4;42,4)	44,3 (36,3;52,6)	50,2 (36,9;63,5)	34,5 (24,8;45,7)	39,9 (33,6;46,6)
3.001,00-8.000,00	29,2 (20,4;39,9)	16,9 (10,3;26,5)	10,5 (5,6;19,0)	3,2 (1,5;6,9)	10,6 (7,9;14)
≥8.001,00	4,8 (2,3;9,9)	1,5 (0,6;4,0)	0,9 (0,3;2,6)	0,0 (0,0;0,0)	1,1 (0,6;1,8)
Ignorado	22,1 (12,0;37,1)	9,1 (4,6;17,0)	1,0 (0,5;2,4)	5,9 (2,3;14,2)	7,0 (4,5;10,7)
**Características da mãe**					
**Faixa etária (anos)**					
<20	1,6 (0,6;4,5)	0,7 (0,2;2,7)	2,0 (1,0;3,8)	1,5 (0,6;3,5)	1,5 (0,9;2,4)
20-34	66,7 (54,2;77,2)	59,3 (47,0;70,6)	70,4 (60,1;79,0)	65,5 (60,4;70,3)	66,0 (61,5;70,2)
≥35	31,2 (21;43,6)	39,4 (28,1;51,9)	27,4 (18,7;38,3)	31,1 (25,8;37)	31,5 (27,1;36,2)
**Escolaridade (anos)**					
0-8	9,0 (4,8;16,5)	14,9 (8,6;24,5)	6,0 (3,5;9,9)	19,7 (13,4;27,9)	13,8 (10,7;17,7)
9-12	5,7 (3,5;9,1)	15,4 (10,2;22,5)	22,6 (14,8;33,0)	28,5 (18,9;40,5)	22,0 (16,7;28,4)
13-15	40,9 (30,2;52,5)	46,5 (35,9;57,4)	60,9 (50,3;70,6)	41,5 (33,6;49,9)	47,7 (42,3;53,0)
≥16	33,1 (24,8;42,7)	21,4 (14,9;29,8)	9,0 (5,6;14,2)	7,7 (4,4;13,2)	13,3 (10,6;16,5)
Ignorado	11,3 (4,5;25,7)	1,9 (0,8;4,3)	1,5 (0,5;4,8)	2,6 (1,1;5,9)	3,2 (1,9;5,3)
**Trabalho remunerado**	54,7 (42,3;66,6)	50,8 (39,5;62,1)	46,6 (37,7;55,8)	40,7 (33,6;48,2)	45,7 (40,7;50,7)
**Média de filhos vivos por mãe**	2,08 (1,98;2,19)	2,13 (2,02;2,24)	2,15 (2,04;2,25)	2,25 (2,14;2,37)	2,15 (2,10;2,21)
**Características da criança**					
**Sexo**					
Masculino	59,9 (51,3;68,0)	51,3 (45,9;56,7)	48,8 (43,8;53,7)	50,9 (44,5;57,3)	51,4 (48,0;54,9)
Feminino	40,1 (32,0;48,7)	48,7 (43,3;54,1)	51,2 (46,3;56,2)	49,1 (42,7;55,6)	48,6 (45,1;52,1)
**Presença de caderneta de vacinação**	98,2 (92,8;99,6)	99,0 (97,1;99,6)	98,5 (96,6;99,3)	98,5 (92,0;99,8)	98,6 (96,7;99,4)
**Uso de serviço privado para a vacinação**	13,2 (8,2;20,6)	9,0 (3,8;19,9)	7,5 (4,3;12,8)	2,5 (1,1;5,8)	6,2 (4,3;8,9)
**Frequenta creche/escola**	41,6 (32,0;51,9)	38,6 (27,8;50,6)	43,6 (30,4;57,7)	59,8 (47,8;70,6)	49,6 (42,3;56,9)

A caderneta de vacinação foi localizada em 98,6% (IC_95_% 96,7;99,4) da amostra, sem diferença entre estratos e municípios. O uso de serviços privados de vacinação ocorreu em 6,2% (IC_95_% 4,3;8,9) das crianças, com maior proporção no estrato A (13,2%, IC_95_% 8,2;20,6) e menor no D (2,5%, IC_95_% 1,1;5,8) (Tabela 1).

Menos da metade era beneficiário do PBF (42,5%; IC_95_% 37,8;47,3), mais frequente no estrato D (50,0%, IC_95_% 42,5;57,4). Renda familiar foi menor ou igual a R$ 1.000,00 em 41,5% (IC_95_% 34,2;49,3), sendo 56,4% (IC_95_% 44,0;68,1) no estrato D e 14,3% (IC_95_% 7,2;26,4) no estrato A. Mãe com idade entre 20-34 anos foram 66,0% (IC_95_% 61,5;70,2), com maior prevalência no estrato C (70,4%, IC_95_% 60,1;79,0) e estrato B (59,3%, IC_95_%47,0;70,6). Quanto à escolaridade, 47,7% (IC_95_% 42,3;53,0) referiram ter de 13-15 anos de estudo, apresentando diferenças entre estratos (60,9% [IC_95_% 50,3;70,6] no estrato C e 40,9% [IC_95_% 30,2;52,5] no estrato A). O trabalho remunerado era realizado por 45,7% (IC95% 40,7;50,7) dos pais ou responsáveis, variando de 40,7% (IC_95_% 33,6;48,2) no estrato D a 54,7% (IC_95_% 42,3;66,6) no estrato A. A média de filhos por mãe foi de 2,15 (IC_95_% 2,10;2,21). As características mais frequentes foram: sexo masculino (51,4%, IC_95_% 48,0;54,9) e frequentar creche/escola (49,6%, IC_95_% 42,3;56,9) (Tabela 1).

Houve redução nas coberturas vacinais nos primeiros 24 meses de vida em todos os municípios e estratos. A menor cobertura vacinal do esquema completo foi no estrato A (ao nascer BCG: 77,9%; 24 meses: vacina contra varicela: 39,2%), e a maior, no estrato B (ao nascer BCG: 91,5%; 24 meses: vacina contra varicela: 48,4%) (Figura 1A). Imperatriz (ao nascer BCG: 90,8%; 24 meses: vacina contra varicela: 33,8%) e Sobral (ao nascer BCG: 86,2%; 24 meses: vacina contra varicela: 33,8%) apresentaram no seguimento as menores coberturas vacinais, sendo inferiores quando comparados ao consolidado dos municípios pesquisados (ao nascer BCG: 87,7%; 24 meses: vacina contra varicela: 43,9%) (Figura 1B).

**Figura 1:** Evolução da cobertura da cascata de vacinas em doses válidas, em crianças nascidas entre 2017 e 2018 em Caruaru, Imperatriz, Sobral e Vitória da Conquista, de acordo com estrato socioeconômico (A) e município de residência (B), 2020-2022 (n=1.847)

A cobertura vacinal completa foi 49,2% (IC_95_% 43,9;54,5), com maior proporção no estrato D (50,6%, IC_95_% 40,8;60,3) e em Caruaru (57,7%, IC_95_% 49,2;65,7) e menor no C (47,1%, IC_95_% 40,4;53,9) e em Imperatriz (34,2%, IC_95_% 28,7;40,1). A maior cobertura (92,8%, IC_95_% 88,4;95,5) foi verificada para a primeira dose da vacina MenC. A menor cobertura foi a da VOP contra a segunda dose da vacina contra rotavírus humano (78,0%, IC_95_% 73,0;82,3) (Figura 2).

**Figura 2:** Cobertura vacinal por doses válidas de imunizantes, segundo estratos socioeconômicos, em crianças nascidas vivas em 2017 e 2018, residentes em Caruaru, Imperatriz, Sobral e Vitória da Conquista (n=1.847)

O desfecho foi verificado mais significativamente em crianças residente em Sobral (OR 4,35; IC_95_% 3,04;6,21), com renda entre R$ 3.001,00-R$ 8.000,00 (OR-a 1,53; IC_95_% 1,02;2,31) e em mães com mais de 1 filho (OR 1,20; IC_95_% 1,11;1,32) (Tabela 2).

**Tabela 2:** Razões de chances brutas e ajustadas (odds ratio, OR) e intervalos de confiança de 95% (IC95%) da vacinação incompleta em crianças nascidas em 2017 e 2018, segundo característica da família, maternas e da criança. Caruaru, Imperatriz, Sobral e Vitória da Conquista, 2020-2022 (n=1.847)

Variáveis	OR bruta (IC95%)	p-valor	OR ajustada (IC95%)	p-valor
Estrato socioeconômico		0,877		-
A	1,44 (1,11;1,87)		-	
B	1,00		-	
C	1,19 (0,92;1,54)		-	
D	1,12 (0,87;1,45)		-	
**Municípios**		<0,001		0,005
Caruaru	1,00		1,00	
Imperatriz	3,07 (2,35;4,01)		2,80 (1,93;3,80)	
Sobral	3,15 (2,41;4,13)		4,35 (3,04;6,21)	
Vitória da Conquista	1,90 (1,46;2,47)		1,63 (1,19;2,23)	
**Características da família**				
**Programa Bolsa Família**		0,699		-
Sim	1,00		-	
Não	1,04 (0,86;1,25)		-	
**Renda familiar mensal (R$)**		<0,001		0,001
≤1.000,00	1,00		1,00	
1.001,00-3.000,00	1,27 (1,03;1,56)		0,98 (0,75;1,28)	
3.001,00-8.000,00	1,96 (1,44;2,65)		1,53 (1,02;2,31)	
≥8.001,00	2,79 (1,46;5,36)		2,06 (0,94;4,48)	
**Características da mãe**				
**Faixa etária ao nascimento da criança (anos)**		0,869		-
<20	1,00		-	
20-34	0,78 (0,37;1,61)		-	
≥35	0,78 (0,37;1,64)		-	
**Escolaridade (anos)**		0,006		0,907
0-8	1,00		1,00	
9-12	1,03 (0,75;1,41)		0,92 (0,65;1,31)	
13-15	1,11 (0,84;1,45)		0,77 (0,55;1,08)	
≥16	1,63 (1,17;2,28)		0,89 (0,57;1,41)	
**Trabalho remunerado**		0,004		0,330
Sim	1,00		1,00	
Não	1,31 (1,09;1,58)		1,25 (0,99;1,58)	
**Filhos vivos por mãe**		<0,001		<0,001
Média	1,17 (1,08;1,26)		1,20 (1,11;1,32)	
**Características da criança**				
**Sexo**		0,479		-
Masculino	1,00		-	
Feminino	1,07 (0,89;1,28)		-	
**Presença de caderneta de vacinação**		0,181		0,261
Sim	1,00		1,00	
Não	2,45 (0,66;9,06)		0,47 (0,12;1,83)	
**Uso de serviço privado para a vacinação**		0,009		0,062
Sim	1,66 (1,14;2,43)		1,33 (0,84;2,12)	
Não	1,00		1,00	
**Frequenta creche/escola**		<0,001		<0,001
Sim	1,72 (1,43;2,07)		1,14 (0,90;1,45)	
Não	1,00		1,00	

As mães ou responsáveis deixaram de vacinar sua criança por decisão própria em 1,2%, em virtude das dificuldades de levar a criança ao posto de vacinação (8,9%). Apesar de terem ido à unidade de saúde, 37,1% das crianças deixaram de ser vacinadas em alguma oportunidade (Tabela 3).

**Tabela 3:** Percepção de pais ou responsáveis sobre vacinação, segundo estrato socioeconômico, de crianças nascidas entre 2017-2018 em Caruaru, Imperatriz, Sobral e Vitória da Conquista, 2020-2022 (n=1.847)

Variáveis	A (%)	B (%)	C (%)	D (%)	**Total (%)**
**Ausência de vacinação infantil por decisão do adult** **o**	3 (0,7)	4 (0,9)	8 (1,7)	8 (1,7)	23 (1,2)
**Dificuldade em levar a criança ao posto de vacinação**	47 (10,4)	37 (8,1)	35 (7,5)	46 (9,8)	165 (8,9)
**Ausência de vacinação mesmo ao levar a criança ao posto de vacinação**	180 (39,8)	161 (35,1)	165 (35,3)	179 (38,2)	685 (37,1)
**Considera vacinas importantes para saúde**					
Não	1 (0,2)	-	-	2 (0,4)	3 (0,2)
Indiferente	3 (0,7)	1 (0,2)	3 (0,6)	2 (0,4)	9 (0,5)
Sim	448 (99,1)	458 (99,8)	465 (99,4)	464 (99,1)	1.835 (99,4)
**Considera desnecessárias vacinas contra doenças erradicadas**					
Sim	53 (11,7)	32 (7,0)	81 (17,3)	69 (14,7)	235 (12,7)
Indiferente	29 (6,4)	32 (7,0)	58 (12,4)	17 (3,6)	136 (7,4)
Não	370 (81,9)	395 (86,1)	329 (70,3)	382 (81,6)	1.476 (79,9)
**Considera vacinas importantes para saúde do bairro**					
Não	2 (0,4)	-	1 (0,2)	5 (1,1)	8 (0,4)
Indiferente	24 (5,3)	26 (5,7)	23 (4,9)	5 (1,1)	78 (4,2)
Sim	426 (94,2)	433 (94,3)	444 (94,9)	458 (97,9)	1.761 (95,3)
**Receio de reações graves**					
Sim	95 (21,0)	93 (20,3)	87 (18,6)	92 (19,7)	367 (19,9)
Indiferente	42 (9,3)	26 (5,7)	65 (13,9)	36 (7,7)	169 (9,1)
Não	315 (69,7)	340 (74,1)	316 (67,5)	340 (72,6)	1.311 (71,0)
**Confiança em vacinas distribuídas pelo governo**					
Não	7 (1,5)	6 (1,3)	6 (1,3)	5 (1,1)	24 (1,3)
Indiferente	12 (2,7)	11 (2,4)	11 (2,4)	15 (3,2)	49 (2,7)
Sim	433 (95,8)	442 (96,3)	451 (96,4)	448 (95,7)	1.774 (96,0)

As vacinas foram consideradas importantes intervenções em saúde para 99,4% das mães ou responsáveis. Na população pesquisada, há relatos de que ‘*considera desnecessárias vacinas contra doenças* erradicadas’ (12,7%), ‘*considera vacinas importantes para saúde do* bairro’ (0,4%), ‘*receio de reações graves*’ (19,9%) e ‘*não confiam nas vacinas oferecidas pelo governo*’ (1,3%) (Tabela 3).

## DISCUSSÃO

Baixa cobertura vacinai foi observada em crianças até dois anos de vida residentes nos quatro municípios investigados no interior do Nordeste brasileiro. A cobertura vacinal completa Imperatriz, em um terço, e em Caruaru, com pouco mais da metade das crianças com esquema vacinal completo. Boa parte das crianças estava, então, suscetível a doenças imunopreveníveis dos municípios estudados.

Não houve alcance da meta recomendada pelo PNI para nenhum dos imunobiológicos avaliados, observando-se comportamento heterogêneo das taxas, sendo mais baixas para os imunizantes multidoses. As poucas exceções foram para aquelas vacinas aplicadas no início do calendário básico, especialmente no estrato socioeconômico mais baixo que alcançou maior cobertura vacinal para vacinas aplicadas ao nascimento. As principais causas de hesitação vacinal referidas refletem questões relacionadas à decisão dos pais contra a vacinação, à restrição do acesso por dificuldades de deslocamento e aos aspectos operacionais nas unidades de vacinação.

Baixas coberturas vacinais encontradas sugerem que os municípios do interior do Nordeste contribuem para o decréscimo e a heterogeneidade que têm sido constatados no país desde 2016,^4,6^ inclusive com altas taxas de abandono, evidenciando-se nos resultados do seguimento em cascata da cobertura vacinal. A complexidade do calendário vacinal infantil com inserção de novas vacinas, as barreiras de acesso por dificuldades de deslocamento e horário de funcionamento das salas de vacinação, a logística geral e infraestrutura insuficientes, a eventual escassez de imunobiológicos e insumos na APS, a hesitação vacinal, a desinformação sobre vacinas e os condicionantes socioculturais foram as principais causas relatadas pelos entrevistados neste inquérito. Estas estão em consonância com os fatores citados na literatura nacional e internacional.^1,6,17-19^

Salienta-se que oportunidades perdidas de vacinação ocorreram em todos os estratos socioeconômicos, principalmente no mais alto. Isso indica a necessidade de desenvolvimento de estratégias contextualizadas às diferentes realidades socioeconômicas para superação deste problema, sendo importante a condução de pesquisas qualitativas para a melhor compreensão de seu processo de determinação.

A associação da vacinação incompleta às mães sem trabalho remunerado e com mais de um filho foi semelhante ao observado em outros estudos realizados no Brasil em décadas anteriores.^10,11^ Isso pode indicar que a situação de vulnerabilidade social é um dos fatores que contribui para a vacinação incompleta e, deste modo não cumprimento do calendário vacinal, contexto já verificado em outros estudos no Brasil e em países de baixa renda.^11,20,21^

Políticas de transferência de renda condicionadas à vacinação infantil mostraram-se efetivas.^22,23^ Esse fato pode ser constatado nesta investigação que revelou coberturas vacinais mais elevadas no estrato socioeconômico mais baixo, no qual encontra-se a maior proporção de beneficiários do PBF. No estrato C, as coberturas vacinais foram abaixo das desejadas. Isso evidencia a existência de maior hesitação vacinal nas populações desfavorecidas que não são estimuladas a cumprir a referida condicionalidade do PBF e que, pelo baixo nível de escolaridade, possivelmente apresentam maior sensação de incertezas e ambiguidades de informações em relação à vacinação.^24^

Apesar de ter sido baixa a proporção de pais ou responsáveis que utilizaram serviços privados de vacinação, essa variável foi associada à incompletude vacinal, provavelmente, em virtude da limitação de informações de dados de seguimento das crianças oriundas dos serviços públicos de vacinação, assim como das crianças dos serviços privados de vacinação que não enviam sistematicamente o registro de doses aplicadas para a vigilância.^11^ A qualificação do seguimento das crianças vacinadas nos serviços privado de vacinação deve ser considerada nas estratégias para resgate das coberturas aos patamares anteriores a 2016. Isso indica a necessidade de diálogo constante com dirigentes dos serviços privados para intercambialidade de dados entre os serviços públicos para que se incorporem ao esforço nacional de proteção de toda a população infantil.^25^

Os resultados advindos com o sucesso do PNI − com a contínua implementação das estratégias de educação, comunicação e informação em saúde junto à sociedade para controlar e eliminar doenças imunopreveníveis, bem como de uma estrutura efetiva e capilarizada das ações de vacinação − não foram mantidos. O contexto das notícias falsas, especialmente relacionadas à situação da covid-19, fortaleceu o crescente movimento negacionista relativo às vacinas nos últimos anos,^1,26^ estimulou a baixa percepção pela população quanto ao risco e à gravidade inerentes ao ressurgimento de doenças imunopreveníveis e, possivelmente, dificultou o alcance das coberturas vacinais desejáveis para o país, incluindo municípios do interior do Nordeste.

Iniciativas vêm sendo adotadas pelo Ministério da Saúde, voltadas ao fortalecimento do PNI^4^ e em articulação com a APS, para expansão das campanhas educativas, ampliação das salas de vacinação e horários de funcionamento nos territórios, considerando especificidades próprias. Tais iniciativas representam esforços para a garantia de sustentabilidade das ações de vacinação no Sistema Único de Saúde, inclusive na perspectiva de novos avanços para melhoria da situação de saúde da população brasileira.

Este estudo apresenta limitações, particularmente pelas questões impostas diante da falta de realização do censo demográfico em 2020, o que impôs o uso de dados de 2010. Este estudo pode apresentar diferenciação para algumas áreas em relação aos dados atuais, considerados para a definição dos estratos socioeconômicos. Acrescentam-se os problemas inerentes a inquéritos domiciliares, em função da desconfiança, insegurança ou desinteresse na participação, especialmente no estrato de melhor poder aquisitivo, que se acentuaram pela necessidade de distanciamento social devido à pandemia de covid-19. Esse problema foi equacionado com ampliação do número de crianças selecionadas em cada estrato e do período de coleta de dados. Acresce-se a ocorrência de dificuldades para leitura das cadernetas de vacinação tanto pela ausência de padronização dos registros, quanto pela pouca legibilidade ou pelos erros de preenchimento. Para minimizar essas dificuldades, a leitura era conduzida por profissionais treinados e, quando necessário, procedia-se à consulta direta ao Sistema de Informação do PNI.

Chama-se atenção para questões operacionais e para a desinformação. Mesmo com todos os problemas identificados, constatou-se consciência em relação à importância da vacinação pela população pesquisada.

Os resultados apresentados neste estudo, em complementação aos demais derivados do inquérito nacional, trazem, em perspectiva à gestão pública em saúde, informações imprescindíveis e estratégicas ao microplanejamento.^27^ Recomenda-se construção de estratégias locais mais custo-efetivas e participativas visando ao desenvolvimento de atividades apropriadas e eficazes de vacinação e ao alcance das metas vinculadas a esses indicadores estratégicos, que refletem diretamente as condições de saúde, especialmente as da população infantil.

Em conclusão, baixas coberturas vacinais e vacinação incompleta foram observadas em crianças particularmente de Sobral, mas também em Caruaru, Imperatriz e Vitória da Conquista. Pessoas com maior renda e mais de um filho demonstraram maior risco para vacinação incompleta. A utilização de serviços privados de vacinação, juntamente com ausência de trabalho remunerado e baixa escolaridade, podem indicar também contextos de vulnerabilidade. A decisão de não vacinação pelos pais ou responsáveis e a limitação do acesso à saúde por dificuldades no deslocamento às unidades de vacinação foram relevantes e tidas como questões relacionadas à hesitação vacinal para a coorte analisada. Incluem-se nesse contexto aspectos operacionais críticos ao funcionamento das salas de vacina no SUS, com significativa expressão para existência de crianças não vacinadas, mesmo estando presente na unidade de vacinação.
